# COVID – 19 related knowledge and preventive practices early in the outbreak among health care workers in selected public health facilities of Illu aba Bor and Buno Bedelle zones, Southwest Ethiopia

**DOI:** 10.1186/s12879-021-06218-0

**Published:** 2021-05-27

**Authors:** Dereje Tsegaye, Muluneh Shuremu, Dereje Oljira, Sileshi Dubale, Getachew Befekadu, Kebebe Bidira

**Affiliations:** College of Health Science, Mettu University, Mettu, Ethiopia

**Keywords:** Knowledge, Practice, COVID-19, Health care workers, Ethiopia

## Abstract

**Background:**

Novel-coronavirus 2019 (COVID-19) disease is currently a worldwide health risk and public health emergency concern. The virus is transmitted from an infected person to another person through close contact and droplets. Frontline health care workers are the most at risk of infection, and so a WHO interim guidance document was issued by the World Health Organization (WHO) which underscores the importance of proper sanitation and waste management practices for COVID- 19 in health-care settings. This study aimed at assessing knowledge and preventive practices towards Covid-19 among health care providers in selected health facilities of Illu Aba Bor and Buno Bedele zones, Southwest Ethiopia.

**Methods:**

An institution-based cross-sectional study was conducted from April to May 2020 among 330 health workers in selected health facilities of Illu Aba Bor and Buno-Bedelle Zones, Southwest Ethiopia. Data were collected using a self-administered structured questionnaire. The collected data were entered into Epidata version 3.1 and exported to SPSS version 23 for analysis. Bivariate and multivariable logistic regression analysis was used to identify independent predictors of preventive practices towards Covid-19. Statistical significance was declared at a *p*-value of < 0.05.

**Result:**

The majority of respondents (93.3%) demonstrated good knowledge of COVID-19, and the mean (SD) knowledge score was 9.04 ± 1.06. **Nearly** two-thirds (64.2%) of the study participants had good infection prevention practices. Being male (AOR = 3.65, 95% CI: (1.96, 6.80)), education level (AOR = 1.82, 95% CI (1.02, 3.22)), profession (AOR = 3.17, 95% CI (1.08, 9.33)), service year (5–10 years) (AOR = 2.00 (1.02, 3.92)) and more than 10 years (AOR = 3.14 (1.51, 6.52)), availability of personal protective equipment (AOR = 1.96 (1.06, 3.61)) and Knowledge level (AOR = 2.61 (1.48, 4.62)) were independent predictors of COVID-19 preventive practices.

**Conclusion:**

The overall level of knowledge of HCWs was good. However, the practice was relatively low. Gender, educational status, profession, year of service, knowledge towards COVID-19, and availability of personal protective equipment were independent predictors of good infection prevention practices. Optimizing the infection prevention and control loop of the health facilities is recommended.

**Supplementary Information:**

The online version contains supplementary material available at 10.1186/s12879-021-06218-0.

## Background

Novel-coronavirus 2019 (COVID-19) disease is currently a worldwide health risk and public health emergency concern [1]. The outbreak was first reported in late December 2019 in Wuhan of China, Hubei Province, when groups of pneumonia cases of unknown etiology were found to be closely related to epidemiologically linked exposure to the seafood market and untraced exposures [2].

According to the World Health Organization (WHO) daily situation report, after the coronavirus disease 2019 outbreak, 22,073 cases were reported to the WHO as of April 2020 among healthcare workers [3]. In early March, this number increased to 3300 and a mimimum of 22 died in China, over 2600 infected with 13 deaths in Italy [4, 5]. Though the disease was initially slow to reach African countries, it’s currently rising exponentially on the continent and is probably going to cause severe illness and deaths [6]. In Ethiopia, there have been 35 confirmed COVID-19 cases since the primary case on 13 March 2020 and then the number increased to 117, as of April 24, 2020 [7, 8].

According to the available evidence, the virus is transmitted from an infected person to another person through close contact and droplets, and so those most at risk of infection are frontline health care workers (HCWs) caring for COVID-19 patients [9, 10].

Evidence shows that proper infection prevention and control (IPC) measures during outbreak management could change the course of the outbreak [11]. However, the present IPC behaviors are sub optimal. A study on the Lassa Fever outbreak among Health care workers (HCWs) showed that none them met the minimum standards of infection prevention practices during the first contact with fever cases [12]. Occurrence of an epidemic, contact with confirmed and suspected cases, key clinical departments (such as ICU and emergency unit) influence the infection prevention and control behaviors and critical risk factors in the pandemic outbreak and always cited as important causes of high healthcare-associated prevalence worldwide [13–15]. years of experience and preparedness are other factors related to healthcare workers’ infection prevention and control behaviors [14].

Owing to the current pandemic, an urgent interim guidance document was issued by the World Health Organization (WHO) which underscores the importance of proper sanitation and waste management practices for COVID-19 in health-care settings [16]. The guideline builds on and further emphasizes the prevailing standard infection prevention and control guidelines for health facilities [17, 18].

Frontline Healthcare workers are at an increased risk of acquiring the virus owing to overcrowding and lack of sanitary facilities which can be compounded by inadequate awareness of some healthcare workers. To the best literature search, few studies were conducted on the extent of awareness and infection prevention practice of healthcare workers. Thus, this study aimed at assessing health care workers’ self-reported knowledge and infection prevention practices towards the VOVID-19.

## Methods

### Study setting and design

This cross-sectional study was carried out in selected public health facilities in Ilu Abba Bor and Buno Bedelle Zones. Ilu Abba Bor zone and Buno Bedelle Zone are out of the 21 zones of Oromia National regional state situated in the southwest of the region and located at a distance of about 600 km and 483 from the center of the region respectively. They cover the western part of the region and lie between 34^0^ 52′12 “E to 41^0^ 34 ‘55” E longitudes and 7^0^ 27′ 40 “N to 9^0^ 02 ‘10” N latitude. Illu Aba Bor Zone has one town administration and 14 rural districts with a projected total population of 1,606,502. One referral and District hospital are found in the zone serving a population of the zone. Buno Bedelle Zone has one town administration and 14 rural districts with a projected total population of 815,437. The zone has three functional hospitals and one under construction, 32 health centers, and 246 health posts. The study was conducted from April 27 –May 10, 2020.

### Population

All health care providers working in service delivery units in selected health facilities in both Illu Aba Bor and Buno Bedelle Zones were the study population.

### Sample size determination and sampling techniques

The sample size was determined by using a single population proportion formula: *n* = (Zα/2) ^2^ p (1-p)/(d) ^2^, where *n* denotes the sample size, Zα/2 is the reliability coefficient of standard error at 5% level of significance = 1.96, (5%) margin of error tolerated, p = proportion of good preventive practice of COVID-19 (50%, since there was no previous study available). Hence, the final sample size calculated was 345 after adjusting for the total health worker population in the two zones. First, health facilities were identified based on their proximity to metropolitan areas and ease of access, and 30% of these facilities were chosen at random. Finally, all health care workers in the selected health facilities were included.

### Data collection tools and procedures

A self-administered structured questionnaire adapted from WHO resources and a review of relevant literature was used to collect the data [10, 19, 20]. The questionnaire was first prepared in English, then translated to the local language (Afan Oromo), and translated back to English by another person who was blinded to the English version to ensure its consistency. The tool was pretested on 5% of the sample selected from health facilities in Illu aba Bor Zone that were not included in the main study and modified based on the pretest observations. The facilitators were given intensive training for two days before the actual data collection.

The knowledge questions had 12 items covering issues such as COVID-19 symptoms, risk conditions, prognosis, modes of transmission and safety, and precautions. The knowledge score was converted into tertiles, with the highest tertile defining “good knowledge” and the two lower tertiles combined defining “poor knowledge.”

The infection prevention practice was assessed using 16 items. The practice was computed by adding the responses, scoring one for each correct answer, and zero otherwise. The practice score was converted into tertile and the highest tertile was used to define “good practice”, while the two lower tertiles combined were labeled as “poor practice”.

### Data processes and analysis

Data were entered onto EpiData version 3.1.0 to control skip patterns and allow double entry and exported to SPSS version 23 for analysis. Recoding, transforming, and re-categorization of some variables were performed to compute some of the analyses. In all analyses of the data, a two-sided p- was used. Independent sample t-test and one-way analysis of variance (ANOVA) were performed to assess any difference in mean knowledge score by demographic characteristics. Binary and multivariable logistic regression analyses were computed to examine the association between dependent and independent variables. The odds ratio with a 95% confidence interval was used to identify the factors associated with good infection prevention and control practices. Multicollinearity between different predictor variables was assessed. The adequacy of the model was checked using the Hosmer and Lemeshow test for goodness of fit.

## Results

### Socio-demographic characteristics of the respondents

A total of 330 HCWs were included in the study, making the response rate 95.7%. More than half (56.1%) of the respondents were between 25 and 34 years of age. Two hundred and three (61.5%) were male participants. More than two-thirds (69.4%) of the respondents were married. Nearly half (47.6%) of the population is protestant, and Oromo is the dominant ethnic group (87.9%).More than half (53.7%) of the respondents were degree holders. One hundred eighty-four (55.8%) of the HCW were nurses and 108 (40.3%) had less than five years of experience (Table 1).

**Table 1:** Socio-demographic characteristics of the Healthcare workers in Illu Aba Bor and Buno Bedelle Zones, Southwest Ethiopia, 2020

Variables	Category	Frequency	Percent
**Age in years**	< 25	84	25.5
	25–35	185	56.1
	> 35	61	18.4
**Sex**	Male	203	61.5
	Female	127	38.5
**Marital status**	Single	96	29.1
	Married	229	69.4
	Others*	5	1.5
**Religion**	Orthodox	109	33.0
	Muslim	57	17.3
	protestant	157	47.6
	Others	7	2.1
**Ethnicity**	Oromo	290	87.9
	Amhara	26	7.9
	Tigray	6	1.8
	Others**	8	2.4
**Educational status**	Diploma	144	43.6
	Degree	176	53.3
	Masters	10	3.03
**Profession**	Physician	29	8.8
	Nurse	184	55.8
	Midwife Nurse	57	17.3
	Health officer	20	6.1
	Lab. Technicians	22	6.7
	Pharmacy professional	18	5.5
**Years of service**	< 5 years	133	40.3
	5–10 years	108	32.7
	> 10 years	89	27.0

* Divorced/Widowed **Wakefata

### Training and availability of hygiene facilities

The study revealed that more than half (59.1%) of the study participants did not receive training related to infection prevention. One hundred seventy-seven (53.6%) of the study participants reported that the institution does not have an infection prevention program and 201 (60.9%) did not have an active infection prevention team. More than half (54.5%) reported that the institution does not have an emerging infectious disease taskforce (dealing with outbreaks) and infection prevention and control guidelines. Nearly two-thirds (65.8%) and 54.5% of the health workers reported the availability of water and soap at their work unit respectively. *Three-fourths (75.2%) of respondents reported the availability of alcohol or hand sanitizer, and two-thirds reported adequate availability of the necessary personal protective equipment (PPE) at their facility.* One hundred ninety (42.4%) of the respondents reported the availability of colored dust bins to segregate medical waste at their work unit. One hundred twenty-eight (38.8%) of the health workers reported that their place of assignment at the time of data collection was at the outpatient department (Table 2).

**Table 2:** Training and availability of hygiene facilities at Health care facilities of Illu Aba Bor and Bunno Bedelle Zones, Southwest Ethiopia, 2020

Variables	Category	Frequency	Percent
**Ever had training on infection prevention**	Yes	135	40.9
	No	195	59.1
**Infection control program at the institution**	Yes	153	46.4
	No	177	53.6
**Infection control team at the facility**	Yes	139	39.1
	No	201	60.9
**Emerging infectious diseases taskforce (dealing with outbreaks) available**	Yes	154	46.5
	No	180	54.5
**IPC policies and guidelines available**	Yes	150	45.5
	No	180	54.5
**Water facility available**	Yes	217	65.8
	No	113	34.2
**Alcohol/hand sanitizer available at the facility**	Yes	248	75.2
	No	82	24.8
**Adequate soap at the work unit**	Yes	150	45.5
	No	180	54.5
**Adequate disinfectants available at the work unit**	Yes	145	43.9
	No	185	56.1
**Sufficient PPE available**	Yes	109	33.0
	No	221	67.0
**Have safety box for sharp disposal**	Yes	243	73.6
	No	87	26.4
**The working unit has a colored dust bin to segregate medical wastes**	Yes	140	42.4
	No	190	57.6
**Currently assigned place (ward)**	OPD	128	38.8
	Laboratory	35	10.6
	Pharmacy	23	7.0
	Medical ward	22	6.7
	Surgical ward	13	3.9
	Pediatric ward	12	3.6
	TB and ART	12	3.6
	MCH	43	13.0
	GYN & OBS	42	12.7

*IPC* Infection prevention and control, *TB* Tuberculosis

*OPD* Outpatient department, *ART* Antiretroviral therapy

*MCH* Mother and child Health, *PPE* Personal protective equipment

### Knowledge towards COVID-19

A greater part (93.3%) of the respondents demonstrated self-reported good knowledge towards COVID-19 and the mean (±SD) knowledge score was 9.0 4 ± 1.06. Three hundred twenty-two (97.6%) correctly answered the mode of transmission of the virus. Almost all (97%) of the respondents correctly answered that COVID-19 is a viral infection and (97.9%) answered that fever, cough, sore throats, and shortness of breath are common symptoms of COVID-19. More than 90% of HCPs were well aware of the route of transmission of the virus. A similar proportion were also aware that frequent handwashing with soap and water or alcohol-based hand rub, and using face masks can help in the prevention of disease transmission. Three hundred and 11 (94.2%) reported that healthcare workers are at a higher risk of infection. Two hundred ninety-one (88.2%) of the respondents correctly identified the isolation period to be 2 weeks. More than three-fourth (79.7%) answered that there is no vaccine available against COVID-19 and (76.1%) stated COVID-19 could be fatal. Less than three-fourths (70.3%) of the respondents indicated antibiotics are not a first-line treatment for the disease (Table 3).

**Table 3:** Knowledge towards COVID-19 among healthcare workers of Illu Aba Bor and Buno Bedelle Zones, Southwest Ethiopia, 2020

Knowledge questions	Category	Frequency	Percent
**COVID-19 is a virus infection**	Yes	320	97
	No	10	3.0
**COVID-19 is transmitted by close contact with the infected person**	Yes	300	90.9
	No	30	9.1
**Know common modes of transmission**	Yes	322	97.6
	No	8	2.4
**The incubation period is 14 days**	Yes	253	76.7
	No	77	23.3
**Fever, cough, sore throats, and shortness of breath are common symptoms of COVID-19**	Yes	323	97.9
	No	7	2.1
**The isolation period is two weeks**	Yes	291	88.2
	No	39	11.8
**COVID-19 vaccine is available on markets**	Yes	67	20.3
	No	263	79.7
**Antibiotics are the first-line treatment**	Yes	98	29.7
	No	232	70.3
**Frequent hands wash with soap and water or alcohol-based hand rub and using face masks can help in the prevention of disease transmission**	Yes	306	92.7
	No	24	7.3
**Patients with underlying chronic diseases are at a higher risk of infection and death**	Yes	302	91.5
	No	28	8.5
**Healthcare workers are at a higher risk of infection**	Yes	311	94.2
	No	19	5.8
**COVID-19 could be fatal**	Yes	251	76.1
	No	79	23.9
**Knowledge**	Poor	22	6.7
	Good	308	93.3
**Mean ± SD knowledge score**			
**Mean ± SD**	9.0 4 ± 1.06		

*SD* Standard deviation

### Infection prevention practice

**Fig. 1:**
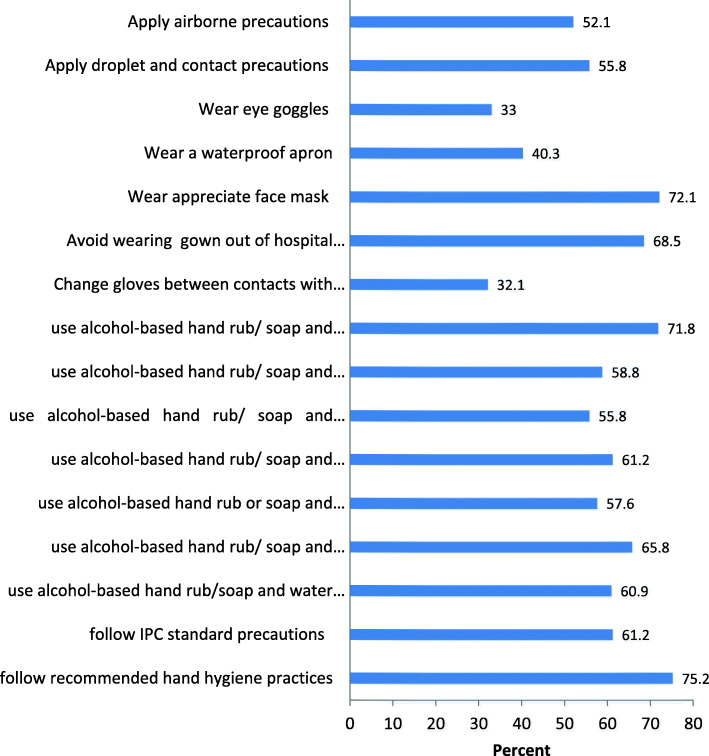
COVID-19 preventive practices among HCWs in Illu Aba Bor and Bunno Bedelle Zones, Southwest Ethiopia, 2020

A summary score was developed from continuous data of the practice of healthcare workers regarding infection prevention towards COVID-19. The mean (±SD) self-reported infection prevention practice was 10.05 ± 4.81. The overall self-reported good infection practice score towards COVID-19 among the healthcare workers was 64.2% (Fig. 1).

### Perceived barriers to infection prevention and control practices

**Fig. 2:**
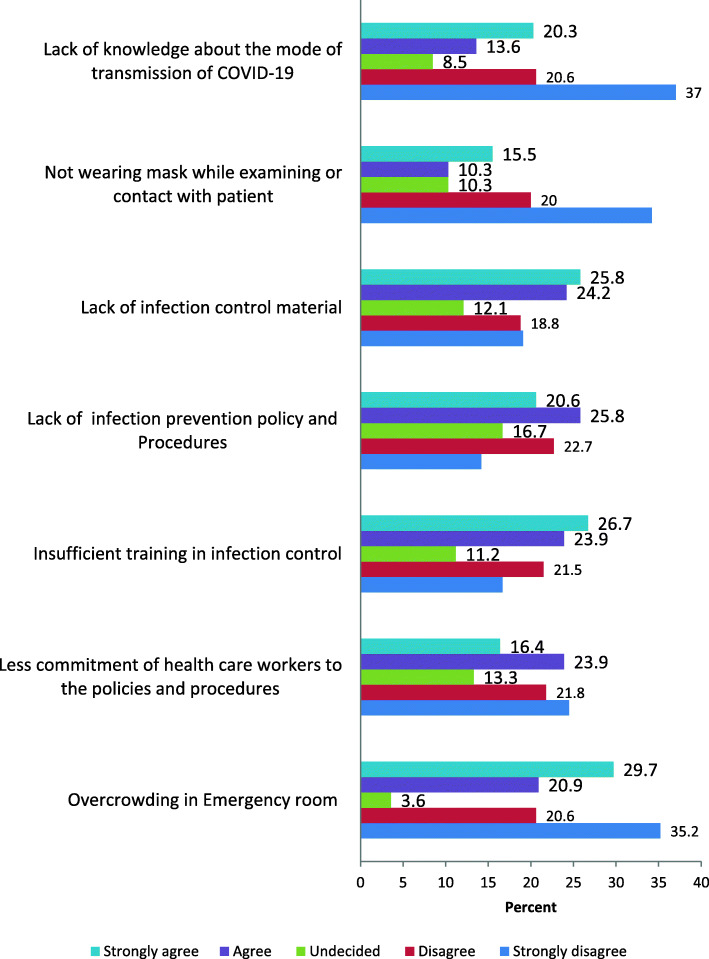
Perceived barriers to infection prevention practice toward COVID-19 by the study participants, Illu Aba Bor and Buno Bedelle Zones, Southwest Ethiopia, 2020

A mixed perception was reported by HCWs regarding barriers to infection prevention and control practices. Ninety-eight (29.7%) of the HCWs presumed that overcrowding in the emergency room was a barrier, whereas, 26.7% strongly agreed that insufficient training on infection prevention was a barrier towards infection prevention and control practices. A quarter (25.8%) of the participants strongly agreed that the limitation of infection prevention equipment was a barrier to infection prevention practices (Fig. 2).

### Differences in knowledge among HCPs towards COVID-19

Independent sample t-test and one-way ANOVA analysis were done to assess the mean knowledge difference between groups regarding socio-demographic characteristics. In both tests, knowledge did not differ significantly (*P* > 0.05) with age, gender, education, experience, or profession (Table 4).

**Table 4:** Test of significance of variation (Independent sample t-test and one way ANOVA) in knowledge score by socio-demographic characteristics, Illu Aba Bor and Buno Bedelle Zones, Southwest Ethiopian, 2020

Variables	Mean(±SD) Knowledge	t-test	F-test	***P***-value
**Age in years**				
**< 25**	8.79 ± 1.27		3.013	0.050
**25–35**	9.13 ± 0.96			
**> 35**	9.08 ± 0.94			
**Sex**				
**Male**	9.05 ± 1.13	1.073		0.284
**Female**	8.93 ± 1.34			
**Educational status**				
**Diploma**	8.96 ± 1.22		0.679	0.508
**Degree**	9.09 ± 0.90			
**Masters**	9.20 ± 1.03			
**Profession**				
**Nurse**	9.01 ± 1.08		0.543	0.744
**Physician**	8.96 ± 1.45			
**Midwife Nurse**	9.11 ± 0.84			
**Health officer**	9.25 ± 0.72			
**Lab. Technicians**	8.83 ± 1.42			
**Pharmacy professional**	9.03 ± 1.05			
**Years of service**				
**< 5 years**	8.89 ± 1.19		2.361	0.096
**5–10 years**	9.18 ± 0.89			
**> 10 years**	9.04 ± 1.05			

*SD* Standard deviation

### Factors associated with infection prevention practice

On multivariable logistic regression analysis, gender, educational status, profession, years of service, knowledge towards COVID-19, and availability of personal protective equipment were significantly associated with good infection prevention practices. As a result, male HCWS were more than three times as good at infection prevention as female HCWS (AOR = 3.65, 95% CI: (1.96, 6.80)).Bachelor degree holders were about two times more likely to practice good infection prevention compared to diploma holders (AOR = 1.82, 95% CI (1.02, 3.22)). Physicians were 3.17 times more likely to practice infection prevention than nurses (AOR = 3.17, 95% CI (1.08, 9.33)). Service year was another factor significantly associated with infection prevention practice. Healthcare workers having a service years of 5–10 years were 2 times more likely to have good infection prevention practice (AOR = 2.00 (1.02, 3.92)) and those who have served for more than 10 years were 3.14 times more likely to have good infection prevention practice (AOR = 3.14 (1.51, 6.52)) compared to those who have served for less than 5 y. Health care workers who had enough supply of personal protective equipment at their work unit were 2 times more likely to have good infection prevention practices compared to those who did not have enough supply of the PPE (AOR = 1.96 (1.06, 3.61)). The knowledge level of health care workers was significantly associated with good infection prevention practices. Health care workers who had a good knowledge score were 2.28 times more likely to have good infection prevention practices compared to those who had poor knowledge scores (AOR = 2.61 (1.48, 4.62)) (Table 5).

**Table 5:** Multivariable logistic regression analysis of factors associated with infection prevention practice among HCWs in Illu Aba Bor and Bunno Bedelle Zones, Southwest Ethiopia, 2020

Characteristics	Category	IPC Practice		COR(95%CI)	AOR(95%CI)
		Good ***N*** (%)	Poor ***N*** (%)		
**Sex**	Male	148 (72.9)	55 (27.1)	3.28 (1.83,5.87)	3.65 (1.96,6.80)**
	Female	64 (50.4)	63 (49.6)	1.00	1.00
**Educational status**	Diploma	82 (56.9)	62 (43.1)	1.00	1.00
	Degree	124 (70.5)	52 (29.5)	1.80 (1.14, 2.86)	1.82 (1.02,3.22)*
	Masters	6 (60.0)	4 (40.0)	1.13 (0.31,4.19)	1.42 (0.31,6.39)
**Profession**	Nurse	109 (59.2)	75 (40.8)	1.00	1.00
	Physician	23 (79.3)	6 (20.7)	0.38 (0.15.0.98)	3.17 (1.08,9.33)*
	Midwife nurse	39 (68.4)	18 (31.6)	0.57 (0.19,1.63)	1.64 (0.77,3.49)
	Health officer	16 (80.0)	4 (20.0)	1.04 (0.25,4.30)	2.42 (0.70,8.37)
	Lab Technicians	16 (72.7)	6 (27.3)	0.69 (0.19,2.55)	0.83 (0.27,2.60)
	Pharmacy professional	9 (50.0)	9 (50.0)	0.26 (0.07,0.95)	0.52 (0.17,1.59)
**Years of service**	< 5	77 (57.9)	56 (42.1)	1.00	1.00
	5–10	75 (69.4)	33 (30.6)	1.65 (0.972.82)	2.00 (1.02,3.92)*
	> 10	60 (67.4)	29 (32.6)	1.51 (0.86,2.64)	3.14 (1.51,6.52)*
**Knowledge**	Poor	10 (45.5)	12 (65.6)	1.00	
	Good	202 (54.5)	106 (34.4)	2.29 (0.96,5.47)	2.35 (1.56,4.98)*
**Training on IPC**	Yes	95 (70.4)	40 (29.6)	1.58 (0.99,2.53)	1.54 (0.87,2.72)
	No	117 (60.0)	78 (40.0)	1.00	1.00
**Active IPC team**	Yes	93 (72.1)	36 (27.9)	1.78 (1.11,2.87)	0.98 (0.52,1.84)
	No	119 (59.2)	82 (40.8)	1.00	1.00
**IPC policy**	Yes	108 (72.0)	42 (28.0)	1.88 (1.18,2.99)	1.21 (0.66,2.35)
	No	104 (57.8)	76 (42.2)	1.00	1.00
**Availability of Water**	Yes	149 (68.7)	68 (31.3)	1.74 (1.09,2.78)	1.26 (0.72,2.20)
	No	63 (55.8)	50 (44.2)	1.00	1.00
**Availability of hand sanitizer**	Yes	168 (67.7)	80 (32.3)	1.81 (1.09,3.02)	0.99 (0.54,1.81)
	No	44 (53.7)	38 (46.3)	1.00	1.00
**Availability of PPE**	Yes	85 (78.7)	23 (21.3)	2.76 (1.62,4.71)	1.96 (1.06,3.61)*
	No	127 (57.2)	95 (42.8)	1.00	1.00

**Significant at < 0.01. *Significant at< 0.05. *IPC* Infection prevention practice

*COR* crude odds ratio, *AOR* adjusted odds ratio, *CI* confidence intervals

*HCW* Health care worker, *PPE* Personal protective equipment

## Discussion

The finding of this study demonstrated that 93.3% of the study participants had self-reported good knowledge towards COVID-19. The finding is consistent with a study in Ho Chi Minh (98.2%) [19], a study in Pakistan (93.2%) [21], and also the finding from China (89%) [22]. Could be due to prolonged exposure to information since it is a global public issue of discussion. Another reason could be the effort of the government and media in providing information beginning from the time of the outbreak.

The study further showed that 64.2% of the study participants had self-reported good infection prevention practices towards COVID-19. This finding is consistent with the findings of a study among nurses in Northern Ethiopia, where 67% practiced good infection prevention against the COVID–19 [23] but less than the finding of a study from Makerere University Teaching Hospitals, Uganda that showed 74% of the study participants demonstrated good practice towards COVID-19 prevention [20]. The possible reason for the difference might be due to variation in the cut of point which is used to determine the outcome variable and variation in type and number of healthcare facilities included in these studies.

The study further revealed that the study participants perceived overcrowding in the emergency room and limited availability of infection prevention material as the major barriers to infection prevention practice. This finding is supported by the study in Pakistan where overcrowding in emergency rooms and limited infection control material were the main barriers in infection control practice [21].

Sex, educational status, occupation, years of service, knowledge, and the availability of personal protective equipment were all found to be significantly associated with good infection prevention practices in multivariable logistic regression analysis.

Male HCWs were more likely to have good infection prevention practices as compared to female health care workers. This study is supported by a study in China that revealed male HCWs promoted IPC behavior compared to females [24].

Healthcare workers having a bachelor’s degrees were more likely to practice infection prevention as compared to diploma holders. The finding of this study contradicts with the study from Makerere University Teaching Hospital, Uganda in which holding a diploma is significantly associated with good practices [20]. This difference can be because of disparities of information among HCWs where doctors involve in searching for information owing to their active roles in improving treatment outcomes of patients with COVID-19.

The health workers’ profession was another factor significantly associated with infection prevention practices. Physicians were more likely to practice infection prevention than nurses. This disparity may be due to differences in knowledge among HCWs where doctors involve in searching for information due to their active roles in improving treatment outcomes of patients with COVID-19. This finding is inconsistent with another study conducted in Pakistan in which Pharmacists were more likely to practice infection prevention practice [21]. This difference might be attributed to the difference in the study setting.

The study further revealed that service year was significantly related to with infection prevention practice. Healthcare workers having longer years of service were more likely to have good infection prevention practices compared to those those that have served for less than 5 y. This finding is in line with the finding of the study conducted in Pakistan that revealed experienced HCWs were more likely to follow precautionary practices [21]. The possible explanation is that experienced workers have skills and knowledge in managing public health emergencies.

Knowledge of the health care workers towards COVID-19 was significantly associated with infection prevention practice. Healthcare workers who had self-reported good knowledge were more likely to have self-reported good practice scores towards COVID-19 than those who had poor knowledge. This finding is comparable to a study finding from Chitwan, Nepal that revealed higher knowledge scores were significantly related to with higher practice scores [25].

Availability of personal protective equipment at the work unit was significantly associated with good infection prevention practices. In contrary to the present study, finding from a study among Orthopedic Surgeons in Wuhan, People’s Republic of China show that insufficient supply of PPE was not associated with Exposures and the COVID-19 Morbidity [26]. This difference could be due to the difference in the supply of personal protective equipment and study settings.

The limitation of this study is that the knowledge level and preventive practice of HCWs may be overestimated, as the HCWs might have answered the questions in a way that they believed was socially acceptable rather than being completely accurate. To make the self-reported compliance closer to the actual, the authors devoted all the staff in the research group and trained carefully, to orient the HCWs to complete the questionnaires based on the actual situation. Lack of adequate similar study also limits comparison of the findings.

## Conclusions

The study revealed that 93.3% of the HCWs had sufficient knowledge of COVID-19 symptoms, risk conditions, prognosis, modes of transmission and safety, and precautions. There was no statistically significant difference in the level of knowledge about COVID- 19 among health care workers for their age, gender, education level professions, or qualifications. Despite the high knowledge level, the preventive practice towards the COVID-19 was relatively low. Overcrowding in the emergency room, insufficient training on infection prevention, and limited availability of infection prevention equipment was the perceived barrier to infection prevention practices. Gender, educational status, profession, year of service, knowledge towards COVID-19, and availability of personal protective equipment were significantly associated with good infection prevention practices. Hence, education intervention and campaigns are required for HCWs to improve their preventive practices, and optimizing the infection prevention and control loop of the health facilities is recommended.

## Supplementary Information

**Additional file 1:.** Annex: Data collection tool/Questionaire

## Data Availability

To keep respondents’ confidentiality, the raw data would not be shared. But, it is available from the corresponding author on reasonable request and the summary data are available in the main document. The tool was attached as an additional file.

## Abbreviations

AOR: Adjusted odds ratio
ART: Anti-Retro Viral
COR: Crude Odds ratio
COVID-19: Corona Virus Disease 2019
FMOH: Federal Ministry of Health
HAI: Healthcare Acquired Infections
HCFs: Health Care Facilities
HCWs: Health Care Workers
IPC: Infection Prevention and Control
MCH: Mother and Child Health
OPD: Out Patient Department
PHEIC: Public Health Emergency of International Concern
PPE: Personal Protective Equipment
SD: Standard deviation
SARS-CoV: Severe Acute Respiratory Syndrome Coronavirus
WASH: Water, Sanitation and Hygiene
WHO: World Health Organizations
