# Supplementary material for: COVID – 19 related knowledge and preventive practices early in the outbreak among health care workers in selected public health facilities of Illu aba Bor and Buno Bedelle zones, Southwest Ethiopia
Source: BMC Infect Dis. 2021 May 27;21:490. doi: 10.1186/s12879-021-06218-0 (PMC8159071; doi:10.1186/s12879-021-06218-0)
Supplement: Supplementary file 1 — Additional file 1:. Annex: Data collection tool/Questionaire [file 12879_2021_6218_MOESM1_ESM.doc]

# ANNEX: DATA COLLECTION TOOL/QUESTIONAIRE

**Section 0: Questionnaire identification**

Date of interview_____________

Name of health facility ___________________

Name of the interviewer: __________________Sign_______________ Date_____

Name of Supervisor_____________________ Sign_______________ Date________

**Section-I: Socio-Demographic characteristics of the respondents**

| **S/N** | **Questions** | **Category** | **Code** | **Skip** |
| --- | --- | --- | --- | --- |
| **Q101** | Facility Type | ____________________________ |  |  |
| **Q102** | Age in years | ____________________________ |  |  |
| **Q103** | Sex | Male  Female | 1  2 |  |
| **Q104** | Marital status | Single  Married  Others(Specify)__________ | 1  2  3 |  |
| **Q105** | Religion | Orthodox  Muslim  protestant  Others(Specify)_______________ | 1  2  3  4 |  |
| **Q106** | Ethnicity | Oromo  Amhara  Tigray  Others(Specify)______________ | 1  2  3  4 |  |
| **Q107** | Educational Lvel | Diploma  Degree  Masters  Others(Specify)__________________ | 1  2  3  4 |  |
| **Q108** | Profession | Physician  Nurse  Midwife Nurse  Health officers  Lab.Tech.  Others(Specify)__________________ | 1  2  3  4  5 |  |
| **Q109** | Years of service | < 5 years  5– 10 years  >10 years | 1  2  3 |  |

**Section-II: Health care workers knowledge regarding COVID-19 and Infection prevention and control**

| S/N | Question | Category | Code | Skip |
| --- | --- | --- | --- | --- |
| **Q201** | COVID-19 is a viral infection | Yes  No | 1  2 |  |
| **Q202** | Incubation period of COVID-19 ( in days) | 1-14  2-7  7-14  7-21 days | 1  2 |  |
| **Q203** | Symptoms of the COVID-19  Infection  *Tick all that apply* | Fever  Cough  Shortness of breath  Diarrhea  Vomiting  Runny nose  Sore throat  Red eyes  Skin rash  Joint or muscle pain  May present with no symptoms | 1  2  3  4  5  6  7  8  9  10  11 |  |
| **Q204** | Mode of transmission | Coughing and sneezing  Hand shaking  Touching surfaces such as doorknobs and tables | 1  2  3 |  |
| **Q205** | COVID-19 is transmitted by close contact with the infected person | Yes  No | 1  2 |  |
| **Q206** | The isolation period is 2 weeks | Yes  No | 1  2 |  |
| **Q207** | COVID-19 vaccine is available on the markets | Yes  No | 1  2 |  |
| **Q208** | Patients with underlying chronic diseases are at a higher risk of infection and death | Yes  No | 1  2 |  |
| **Q209** | Healthcare workers are at a higher risk of infection | Yes  No | 1  2 |  |
| **Q210** | COVID-19 patients develop severe acute respiratory illness? | Yes  No  I do not know | 1  2  3 |  |
| **Q211** | COVID-19 could be fatal | Yes  No | 1  2 |  |
| **Q212** | Antibiotics are first line treatment? | Yes  No | 1  2 |  |

**Section-III: training and availability of hygienic facilities**

| **S/N** | **Questions** | **Category** | **Code** | **Skip** |
| --- | --- | --- | --- | --- |
| **Q301** | Have you ever taken training on infection prevention | Yes  No | 1  2 |  |
|  | Do you have infection control program at your institution? | Yes  No  I don’t know | 1  2  3 |  |
| **Q302** | At your institution, do you have active infection control team? | Yes  No  I don’t know | 1  2 |  |
|  | Do you have an emerging infectious diseases taskforce (dealing with outbreaks)? | Yes  No  I don’t know |  |  |
| **Q303** | Do you have infection control policies and guidelines in your  working department | Yes  No  I don’t know | 1  2 |  |
| **Q304** | What is your IPC role: | I am part of my facility’s IPC team  I am part of my facility’s IPC committee I am my facility’s IPC focal person and there is no IPC team at my facility  Other, please specify__________ | 1  2  3  4 |  |
| **Q305** | Does your working department have water facility | Yes  No | 1  2 |  |
| **Q306** | Do you have alcohol/hand sanitizer in your working unit | Yes  No |  |  |
| **Q307** | Is there adequate soap at your working unit | Yes  No | 1  2 |  |
| **Q308** | Is there adequate disinfectants available in your working unit | Yes  No | 1  2 |  |
| **Q309** | Do you get necessary  personal protective equipment (PPE) such as gloves, apron, goggles and mask when needed | Yes  No | 1  2 |  |
| **Q310** | Does your working unit have colored coded dust bin to segregate medical wastes | Yes  No | 1  2 |  |
| **Q311** | Is there a shortage of staff in your facility | Yes  No | 1  2 |  |
| **Q312** | Currently assigned  places(wards) | Outpatient department  Laboratory Room  Inpatient and Emergency  Medical ward  Surgical ward  TB and ART clinic  MCH department  Gynecology and obstetrics ward  Others(Specify)__________________ | 1  2  3  4  5  6  7  8  9 |  |

| **Section-IV: Compliance/practice of infection prevention and control measures** | | | | |
| --- | --- | --- | --- | --- |
| S/N | Question | Category | Code | Skip |
| Q401 | Do you follow IPC standard precautions when in contact with any patient? | Always, as recommended  Some time  Never | 1  2  3 |  |
| Q402 | Do you use alcohol-based hand rub or soap and water between patient contacts? | Always, as recommended  Some time  Never | 1  2  3 |  |
| Q403 | Do you use alcohol-based hand rub or soap and water before clean or aseptic procedures? | Always, as recommended  Some time  Never | 1  2  3 |  |
| Q404 | Do you use alcohol-based hand rub or soap and water before touching a patient? | Always, as recommended  Some time  Never | 1  2  3 |  |
| Q405 | Do you use alcohol-based hand rub or soap and water after touching a patient? | Always, as recommended  Some time  Never | 1  2  3 |  |
| Q406 | Do you use alcohol-based hand rub or soap and water after touching a patient’s surroundings? | Always, as recommended  Some time  Never | 1  2  3 |  |
| Q407 | Do you use alcohol-based hand rub or soap and water immediately after removal of gloves? | Always, as recommended  Some time  Never | 1  2  3 |  |
| Q409 | Do you protect yourself against body fluids of all patients regardless of their diagnosis? | Always, as recommended  Some time  Never | 1  2  3 |  |
| Q409 | Do you wear clean gloves whenever there is a possibility of exposure to any body fluids? | Always, as recommended  Some time  Never | 1  2  3 |  |
| Q410 | Do change gloves between contacts with different patients? | Always, as recommended  Some time  Never | 1  2  3 |  |
| Q411 | Do you avoid wearing gown out of hospital compounds? | Always, as recommended  Some time  Never | 1  2  3 |  |
| Q412 | Do you wear face mask whenever there is possibility of body fluid/droplet? | Always, as recommended  Some time  Never |  |  |
| Q413 | Do you wear a waterproof apron whenever there is a possibility of body fluid? | Always, as recommended  Some time  Never | 1  2  3 |  |
| Q414 | Do you wear eye goggles whenever there is a possibility of body fluid splashing? | Always, as recommended  Some time  Never | 1  2  3 |  |
| Q415 | Do you apply droplet and contact precautions before entering the room where suspected or confirmed respiratory (COVID-19) patients are admitted? | Always, as recommended  Some time  Never | 1  2  3 |  |
| Q416 | Do you apply airborne precautions for aerosol-generating procedures(e.g. tracheal intubation, non-invasive ventilation, tracheotomy, cardiopulmonary  resuscitation, manual ventilation before intubation, and collection of nasopharyngeal  Swap/aspirate)? | Always, as recommended  Some time  Never | 1  2  3 |  |

**Section-**V: Barriers Perceived by Health Care Professionals

| S/N | Barriers to infection control practice | **Strongly disagree** | **Disagree** | **Undecided** | **Agree** | **Strongly agree** |
| --- | --- | --- | --- | --- | --- | --- |
| Q501 | Overcrowding in Emergency room |  |  |  |  |  |
| Q502 | Less commitment of health care workers to the policies and procedures |  |  |  |  |  |
| Q503 | Insufficient training in infection control measurements |  |  |  |  |  |
| Q504 | Lack of policy and Procedures of infection control Practice |  |  |  |  |  |
| Q505 | Limitation of infection control material |  |  |  |  |  |
| Q506 | Not wearing mask while examining or contact with the patient |  |  |  |  |  |
| Q507 | Lack of knowledge about the mode of transmission of the disease COVID-19 |  |  |  |  |  |
